# Performance assessment across different care settings of a heart failure hospitalisation risk-score for type 2 diabetes using administrative claims

**DOI:** 10.1038/s41598-022-11758-9

**Published:** 2022-05-11

**Authors:** Alessandro Guazzo, Enrico Longato, Mario Luca Morieri, Giovanni Sparacino, Bruno Franco-Novelletto, Maurizio Cancian, Massimo Fusello, Lara Tramontan, Alessandro Battaggia, Angelo Avogaro, Gian Paolo Fadini, Barbara Di Camillo

**Affiliations:** 1 Department of Information Engineering, University of Padova, 35122 Padua, Italy; 2 Department of Medicine, University of Padova, 35128 Padua, Italy; 3 Scuola Veneta di Medicina Generale (SVEMG), Padua, Italy; 4 Società Italiana di Medicina Generale e delle Cure Primarie (SIMG), Florence, Italy; 5 Arsenàl.IT, Veneto’s Research Centre for eHealth Innovation, 31100 Treviso, Italy; 6 Department of Comparative Biomedicine and Food Science, University of Padova, 35020 Legnaro, PD Italy

**Keywords:** Heart failure, Diabetes complications, Type 2 diabetes, Biomedical engineering

## Abstract

Predicting the risk of cardiovascular complications, in particular heart failure hospitalisation (HHF), can improve the management of type 2 diabetes (T2D). Most predictive models proposed so far rely on clinical data not available at the higher Institutional level. Therefore, it is of interest to assess the risk of HHF in people with T2D using administrative claims data only, which are more easily obtainable and could allow public health systems to identify high-risk individuals. In this paper, the administrative claims of > 175,000 patients with T2D were used to develop a new risk score for HHF based on Cox regression. Internal validation on the administrative data cohort yielded satisfactory results in terms of discrimination (max AUROC = 0.792, C-index = 0.786) and calibration (Hosmer–Lemeshow test *p* value < 0.05). The risk score was then tested on data gathered from two independent centers (one diabetes outpatient clinic and one primary care network) to demonstrate its applicability to different care settings in the medium-long term. Thanks to the large size and broad demographics of the administrative dataset used for training, the proposed model was able to predict HHF without significant performance loss concerning bespoke models developed within each setting using more informative, but harder-to-acquire clinical variables.

## Introduction

According to the World Health Organization, cardiovascular diseases (CVDs), a group of disorders affecting the heart and circulatory system, are the leading cause of death globally, taking ~ 18 million lives each year^1^. People affected by chronic diseases are more exposed to CVDs^2^. Particularly, previous studies have shown that diabetes is the 3^rd^ most frequent comorbidity in hospital admissions due to CVD and that people with type 2 diabetes (T2D) have a threefold higher risk of developing CVD, compared to healthy individuals^3^. Among all cardiovascular endpoints in the diabetic population, heart failure (HF) is one of the most relevant, on account of its severity, progressive nature, and recurrence, driving high rates of hospitalisation.

According to randomised controlled trials, one class of glucose-lowering medications used for the management of T2D reduces the risk of hospitalisation for HF (HHF). The use of sodium-glucose co-transporter 2 inhibitors (SGLT2i) has been consistently associated with lower HHF rates in trials and observational studies^4–6^. Yet, due to cost constraints, prescription of such medications is conditioned to a certain clinical spectrum of disease and concomitant treatments. Thus, a rationale allocation of the available resource is critical.

An accurate prediction of HF occurrence could aid the selection of the most appropriate therapeutic approach for T2D, as it may allow caregivers to more easily stratify patients according to their risk, to improve patient quality of life and helping healthcare providers meet higher quality standards of care. Moreover, HF prediction tools may be useful for payers and administrators, since being able to identify high-risk patients and reasonably allocating resources towards their care may result in reduced morbidity, hospitalisation rates, mortality, and hence great cost savings^7^.

Several CVD risk scores for the diabetic population have been proposed in the literature. Often, such scores would have been developed for a given population, or care setting, and, thus, cannot be directly used in a different scenario than the one used for model training before external validation^8,9^. Input data variability is also a factor to be considered, as the vast majority of risk scores rely on the acquisition of specific sets of clinical data. E.g., the UK Diabetes Prospective Study (UKPDS) risk engine^10^, widely used in the literature to predict CVDs, identified predictors such as glycated haemoglobin (HbA1c) levels, systolic blood pressure, and total cholesterol/HDL ratio. Similarly, the WATCH-DM risk score^11^ used BMI, creatinine, fasting plasma glucose, and QRS duration among other predictors. The clinical information needed to implement these risk scores, however, may not be readily available with adequate coverage at the Institutional level^12^, resulting in a narrower userbase and excluding relevant stakeholders such as administrators and payers. On the contrary, being able to properly stratify the population of people with diabetes based on predicted CVD risk is instrumental for healthcare systems to better allocate financial resources and reduce costs of care.

Motivated by the current lack of a simple HHF risk assessment tool relevant to both caregivers and payers (the former need to prescribe the correct medication, the latter need to have state-of-the-art tools to evaluate economic impact vs. quality of life gains), in this study, a dataset of administrative claims collected from more than 175,000 individuals with T2D was used to develop a point-based HF hospitalisation (HHF) risk score based on Cox regression coefficients. First, the underlying Cox model was compared to the point-based risk score to confirm that the two were indeed equivalent in terms of performance. Then, to verify that it could be successfully applied across different care settings, the proposed risk score was externally tested on two datasets, one coming from a diabetes outpatient clinic, and the other from a network of primary care general practitioners^13^. Finally, to quantify the impact of clinical data (or the lack thereof) on prediction performance, the HHF risk score was compared to ad hoc Cox models developed using all the medical, laboratory, and lifestyle data that were available in the diabetes outpatient clinic and primary care datasets.

## Methods

### Datasets

Three anonymised datasets were used for the analysis. The first was an administrative claims dataset, similar to typical datasets commonly used for large-scale observational studies on diabetes^14^, containing information on demographics, exemptions from co-payment, filled prescriptions, outpatient visits, specialist referrals, and diagnoses at hospital discharge concerning the entire Veneto region (North-East Italy, ~ 5 million inhabitants). The second dataset was collected at the diabetes outpatient clinic of the University Hospital of Padova (Padova, Italy) and contained all information usually available in a hospital setting (e.g., vital signs, anthropometric measures, physiological parameters, laboratory test results, known comorbidities, current, and past therapy). Finally, the third dataset was obtained from a primary care database (MilleinRete) maintained by general practitioners affiliated to the Venetian School of General Practice (SVEMG) and included all information usually available in a primary care setting (e.g., prescribed medications, specialist visits, laboratory test results, current, and past pathologies)^15^.

The three datasets were harmonised to consider only citizens of the Veneto Region affected by T2D (based on reported diagnosis or previously validated algorithm^16^), observed between January 1st, 2011, and September 30th, 2018. HHFs were identified using ICD-9-CM diagnosis code 428^17^; if a patient had more than one HHF only the first one was considered. Patients with HF before the start of the observation period were excluded from all datasets, patients treated with diuretics not belonging to ATC^18^ classes C03A or C03B (classes associated with low-ceiling diuretics) were excluded from the administrative claims and diabetes outpatient clinic datasets. Patients treated with diuretics of any class were excluded from the primary care dataset as sufficiently detailed ATC class information was not available. These exclusions were performed to predict HHF without any evidence of latent HF at baseline^19^. After the application of the exclusion criteria, 176,018 patients remained in the administrative claims dataset (median follow-up: 65 months; IQR: 38–68), 3811 patients in the diabetes outpatient clinic dataset (median follow-up: 47 months; IQR: 26–75), and 5849 patients in the primary care dataset (median follow-up: 73 months; IQR: 32–92). Note that the three datasets, though collected within the same healthcare system, could not be linked, because subjects were anonymised, and the data gathering process was different for each dataset. The same subject is thus likely to be represented differently in the different datasets.

As part of the harmonisation process, the diabetes outpatient clinic and primary care datasets were processed to obtain a set of 27 common variables overlapping with those available in the administrative claims dataset. The final set of common predictors for all datasets comprised 24 dichotomous variables related to prescribed medications, sex, and concomitant illnesses or comorbidities; and 2 continuous variables related to age and diabetes duration. Clinical variables related to raw laboratory biomarkers and lifestyle (namely: total cholesterol/HDL ratio, HbA1c, estimated glomerular filtration rate (eGFR)^20^, BMI, blood pressure, microalbuminuria, ever smoker, alcohol, and physical activity) were also available in the diabetes outpatient clinic and primary care datasets, but not in the administrative claims dataset. All variables were collected in the year before the index date, defined as the first prescription of medications used in diabetes (ATC class A10). Quantitative laboratory measurements, when available, were obtained, due to data acquisition constraints, as the average of the values measured during that year.

This study was conducted in accordance with the principles of the Declaration of Helsinki, in compliance with national regulations on retrospective studies using routinely accumulated data (Italian Medicines Agency, “Agenzia Italiana del Farmaco,” determination 20/03/2008), the study protocol was notified to the ethical committee of the University Hospital of Padova (prot. 75856 dated 18/12/2019) and a protocol-specific consent was waived. Italian Medicines Agency determination 20/03/2008 grants tacit approval 60 days after submission of the study protocol. All patients had provided informed consent to the re-use of medical data for research purposes as a prerequisite for entering the databases.

### Data pre-processing

The administrative claims dataset was randomly divided into three subsets: training (146,018 patients), validation (15,000), and test (15,000). The diabetes outpatient clinic and primary care datasets, more modest in size, were randomly divided only into two subsets: training (diabetes outpatient clinic: 2811; primary care: 4849) and test (diabetes outpatient clinic: 1000; primary care: 1000). The operative function of the validation test was substituted by cross-validation^21^. The post index-date follow-up observation was truncated at 5 years.

The datasets were independently pre-processed according to the following pipeline:

- Nonnormally distributed continuous variables were log-transformed after assessing normality via visual inspection of quantile–quantile plots.
- All continuous variables were standardised by subtracting the mean and dividing by the standard deviation.
- Continuous variables with missing data were imputed using the MICE algorithm^22^; dichotomous variables needed no imputation (either because they were always known, e.g., sex; or because they conformed to the “absence of evidence is evidence of absence” hypothesis, e.g., medications).
- Collinear variables were iteratively excluded as per the Variance Inflation Factor (VIF)^23^ until all VIFs were < 3. The threshold level was chosen between 2 and 3 (no variables excluded with 4 and 5) the latter being preferred so as to exclude collinear variables while retaining known predictors of HHF such as diabetes duration.
- Variables with intra-class variance close to 0 were excluded as per the convergence requirements of the Cox model.

All data-dependent parameters needed for the pre-processing were estimated only on the training sets.

### Model and score development strategy

All models developed in this study were based on Cox regression^24^. The Cox model was preferred to other methodological approaches for two main reasons: (1) is easy to obtain a simple point-based score from its coefficients, and (2) it emerged as a well-performing and certainly parsimonious alternative from previous studies on HHF prediction in diabetes^25^. Feature selection was performed using the forward recursive feature selection (FRFS) approach^26^, treating the optimal set of features for each configuration as a hyper-parameter. The only other hyper-parameter was the L2 regularisation strength of the Cox model, α, which was randomly sampled from a log-uniform distribution ranging from ${10}^{-7}$ to ${10}^{-1}.$ Hyper-parameters were optimized using a random search approach^27^ considering 500 possible values of α for each FRFS iteration. The best hyper-parameters (i.e., α and the subset of most important features) were chosen as those that maximised the 5-year Harrell’s concordance index (C-index) on the validation set for the administrative claims dataset and the cross-validated C-index for the smaller diabetes outpatient clinic and primary care datasets. For each iteration of the forward recursive feature selection 500 different models must be trained, one for each value of the hyperparameter α. On top of this, the process must be repeated for each cross-validation fold. In view of such a complex training workflow, the number of folds for the cross-validation was set to 5 instead of the most commonly used value of 10. The early stopping criterion for FRFS was a relative variation of the C-index $<0.01\%$. See "Performance evaluation strategy" section for further details on the evaluation metrics.

To make the final model more accessible and easier to use in everyday practice, a point-based risk score was developed to estimate the 5-year risk of HHF using regression coefficients from the administrative Cox model and an age-standardized points scoring system as proposed by Sullivan et.al.^28^. Three risk groups were then identified by dividing the range of possible point-based risk scores (from − 5 to 83) into tertiles. For each group, the corresponding 5-year HHF risk was also computed by dividing the number of subjects belonging to a given group with HHF at 5 years by the total number of subjects belonging to that group.

While the resulting point-based HHF score is the primary output of this work, its nature as an approximation of the linear part of a survival model also allows to convert it back to a probability as per^28^, i.e., by plugging it into the following formula:

1 $$ 5\;year\;HHF\;probability = 1 - S_{0} \left( t \right)^{{\exp \left( {score\left( X \right)*B + age\_midpo{\text{int}} *\beta_{age} - \mu_{age} *\beta_{age} } \right)}} $$

where:

- $S_{0} \left( t \right)$ is the baseline survival function estimated by the underlying Cox model.
- $score\left( X \right)$ is the HHF score associated with the set of individual covariates X.
- $B$ is the number of regression units that will correspond to one point, fixed to $\beta_{age}$ in this case.
- $age\_midpoint$ is the age value that was considered as reference (43 years).
- $\beta_{age}$ is the estimated coefficient associated with age in the underlying Cox model divided by the standard deviation of age computed on the administrative claims training set.
- $\mu_{age}$ is the average age computed on the administrative claims training set.

Note that this transformation does not affect the score’s discrimination ability.

### Performance evaluation strategy

#### Discrimination performance metrics

Discrimination was assessed using both the time-dependent area under the receiver-operating characteristics curve (AUROC)^29^ and Harrell’s C-index^30^, with their 95% confidence intervals^31^. The tests proposed by DeLong et.al.^32^ and by Kang et.al.^33^ were also performed to assess statistically significant differences in performance at the 0.05 level for AUROC and C-index respectively. To investigate possible differences in long- and short-term prediction performance all metrics were calculated on the relevant test sets at five different prediction horizons (PH), ranging from 1 to 5 years (note: the 5-year PH is consistent with the training process), following the approaches detailed in^29^ and^34^. While AUROC and C-index convey similar information on how well a model predicts future outcomes, they differ in the way they treat the ground truth: the AUROC only distinguishes between events and non-events at a certain PH (excluding the subjects who were censored before the PH), while the C-index also takes into account the order of events up until the PH, and all censored patients.

#### Discrimination analysis

The objectives of this analysis were: (1) to assess the performance of the point-based HHF risk score on the administrative test set both in absolute terms and as compared to the underlying Cox model; (2) to verify the applicability of the point-based HHF score to the diabetes outpatient clinic and primary care contexts; and (3) to compare the proposed score, developed using administrative data, to ad hoc models that are directly trained in the diabetes outpatient clinic and primary care contexts, including clinical-level parameters and laboratory data uniquely available in those datasets.

**Figure 1:**
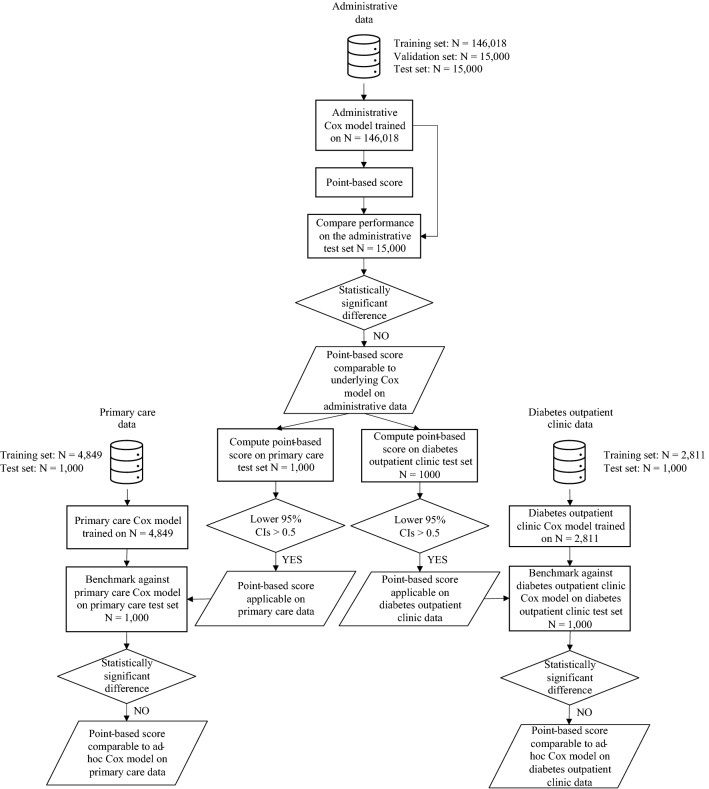
Flowchart of the analysis workflow. The available datasets are represented with the data icon, key decision points are highlighted with the lozenge shape, and conclusions are highlighted with the parallelogram shape.

Hence, first, we computed the point-based HHF score and the predicted probabilities of the underlying Cox model for the subjects of the administrative test set and compared the resulting AUROC and C-index. This analysis was deemed to be successful if the appropriate statistical tests (DeLong and Kang respectively) were not able to detect statistically significant differences between the HHF score and the Cox model discrimination. Second, we computed the HHF score on the harmonised diabetes outpatient clinic and primary care test sets and evaluated its discrimination performance. This analysis was deemed to be successful if all lower bounds of the 95% confidence intervals remained above the random-predictor threshold of 0.5. Third, we compared the HHF score to the ad-hoc models developed on the diabetes outpatient clinic and primary care training sets using all available variables. This analysis was deemed successful if the appropriate statistical tests (DeLong and Kang) were not able to detect statistically significant differences between the HHF score and the ad-hoc models discrimination on the corresponding test sets. The detailed flowchart of the analysis workflow is shown in Fig. 1.

#### Calibration analysis

To complement discrimination, calibration was assessed via the visual inspection of calibration plots (observed HHF risk probability vs. predicted HHF risk probability)^35^ and the Hosmer–Lemeshow statistical test with a significance level set to 0.05^9^.

As all developed models were trained on training sets with a PH fixed to 5 years, recalibration techniques were used to obtain probabilities from 1 to 4 years. The probabilities obtained from both the Cox model and the conversion to the probability of the HHF score as per Eq. (1), were recalibrated using linear recalibration^36^ based on the observed incidence and average predicted HHF risk computed on the training set. Since the 1-to-5-year HHF cumulative incidence was significantly different among the different datasets (see Table 1), the probabilities obtained from the conversion to the probability of the HHF score for the diabetes outpatient clinic and the primary care test sets were recalibrated using parameters estimated from the respective training sets to obtain values comparable with those obtained from the Cox models developed specifically for these settings. Note that the recalibration process does not affect discrimination in any way since the linear transformation employed was strictly monotonic.

**Table 1:** Set of common possible predictors characteristics.

	Administrative claims	Diabetes outpatient clinic	Primary care
N. subjects	176,018	3811	5849
Age (years)	67.6 ± 11.5	67.4 ± 11.1	65.8 ± 12.7
Female sex	73,312 (41.7%)	1548 (40.6%)	2504 (42.8%)
Diabetes duration (years)	6.1 ± 5.4	10.4 ± 9.1	4.3 ± 4.1
Cancer	20,432 (11.6%)	190 (5.0%)	691 (11.8%)
Anaemia	1059 (0.6%)	535 (14%)	298 (5.1%)
Peripheral arterial disease	899 (0.5%)	259 (6.8%)	767 (13.1%)
Chronic kidney disease	1291 (0.7%)	557 (14.6%)	671 (11.5%)
Chronic pulmonary disease	21,705 (12.3%)	151 (4%)	902 (15.4%)
Treated dyslipidaemia	92,990 (52.8%)	2,797 (73.4%)	4083 (69.8%)
Complications renal	113 (0.06%)	1,219 (32%)	12 (0.2%)
Infarction	2249 (1.3%)	237 (6.2%)	194 (3.3%)
Ischemic heart disease	12,034 (6.8%)	302 (7.9%)	550 (9.4%)
Stroke or TIA	4074 (2.3%)	43 (1.1%)	781 (13.4%)
Systemic inflammatory disease	2780 (1.6%)	263 (6.9%)	202 (3.5%)
Calcium channel blockers	41,178 (23.8%)	1058 (27.8%)	1340 (22.9%)
Beta blockers	46,286 (26.3%)	1002 (26.3%)	1340 (22.9%)
Acarbose	2706 (1.5%)	12 (0.3%)	56 (0.9%)
Dpp4i	14,400 (8.2%)	516 (13.5%)	58 (1.0%)
ACE inhibitors	111,619 (63.4%)	1598 (41.9%)	2370 (40.5%)
Insulin	31,978 (18.2%)	1091 (28.6%)	929 (15.9%)
Anticoagulants	17,086 (9.7%)	172 (4.5%)	193 (3.3%)
Pioglitazone	9736 (5.5%)	64 (1.7%)	135 (2.3%)
Ezetimibe	1543 (0.9%)	210 (5.5%)	17 (0.3%)
Sulfonylureas	70,300 (40.0%)	1106 (29%)	1027 (17.6%)
Metformin	139,132 (79.0%)	2933 (77%)	4050 (69.2%)
Platelet aggregation inhibitors	67,212 (38.2%)	1760 (46.2%)	2175 (37.2%)
1-year HHF	1538 (0.9%)	41 (1.1%)	7 (0.1%)
2-years HHF	3166 (1.8%)	88 (2.3%)	20 (0.3%)
3-years HHF	4754 (2.7%)	132 (3.5%)	41 (0.7%)
4-years HHF	6305 (3.6%)	172 (4.5%)	66 (1.1%)
5-years HHF	7773 (4.4%)	211 (5.5%)	84 (1.4%)

Continuous variables are presented as mean ± standard deviation, binary variables as count (percentage relative to N. subjects).

## Results

### Cohorts description

A set of pseudo-clinical variables was derived from the administrative claims dataset following a process described before^12^. Table 1 shows the common predictors for the three available datasets (see Supplementary Table SI for a comparison between variable distributions in the training, validation, and test splits of the administrative dataset, demonstrating that they are representative of each other). Women were equally underrepresented relative to men between the three datasets (~ 41%/59), patients enrolled at the diabetes outpatient clinic had a much longer history of diabetes when compared to patients belonging to the other two care settings (~ 10.4 years vs. 6.1 administrative and 4.3 primary care), and patients under primary care were, on average, approximately 2 years younger than those in the other datasets (65 years vs. 67 years administrative and 67 diabetes outpatient clinic).

### Variables selected as predictors of HHF

**Figure 2:**
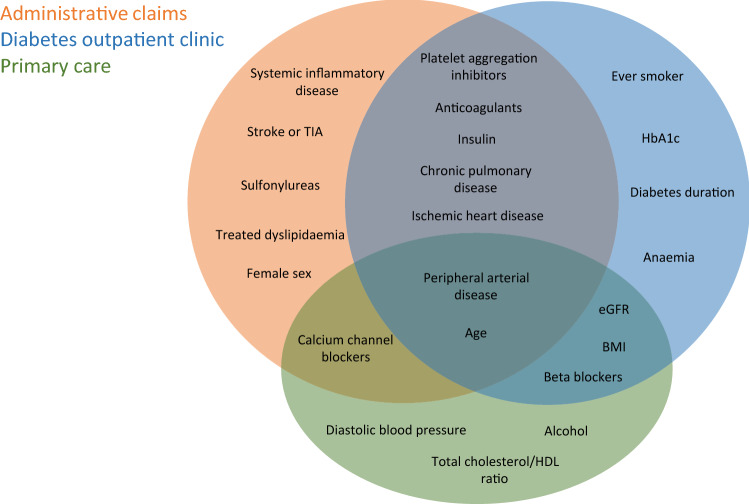
Sets of variables selected for each developed model. Variables selected for the administrative model are highlighted in the orange set, variables selected for the diabetes outpatient clinic are highlighted in the blue set and variables selected for the primary care model are highlighted in the green set. Variables that are selected in more than one model are represented inside the intersection among the different sets.

Variable selection was conducted as part of the hyperparameter tuning phase of the training process. Thirteen out of 27 variables were selected for the administrative claims Cox model, namely: age, platelet aggregation inhibitors, anticoagulants, calcium channel blockers, insulin, female sex, chronic pulmonary disease, treated dyslipidaemia, sulfonylureas, ischemic heart disease, stroke or TIA, peripheral arterial disease, and systemic inflammatory disease. Of the fourteen variables selected for the diabetes outpatient clinic Cox model, ten were among those available also at the administrative level (age, platelet aggregation inhibitors, chronic pulmonary disease, ischemic heart disease, peripheral arterial disease, insulin, anaemia, anticoagulants, diabetes duration, and beta-blockers) and four were clinical (HbA1c, eGFR, BMI, and ever smoker). Of the nine variables selected for the primary care Cox model, four were among those available also at the administrative level (age, peripheral arterial disease, beta-blockers, and calcium channel blockers) and five were clinical (eGFR, BMI, diastolic blood pressure, alcohol, and total cholesterol/HDL ratio). Figure 2 shows the selected features for each developed model (administrative, diabetes outpatient clinic, and primary care).

### Discrimination analysis

#### Discrimination of the point-based HHF risk score and the underlying cox model in the administrative test set

**Figure 3:**
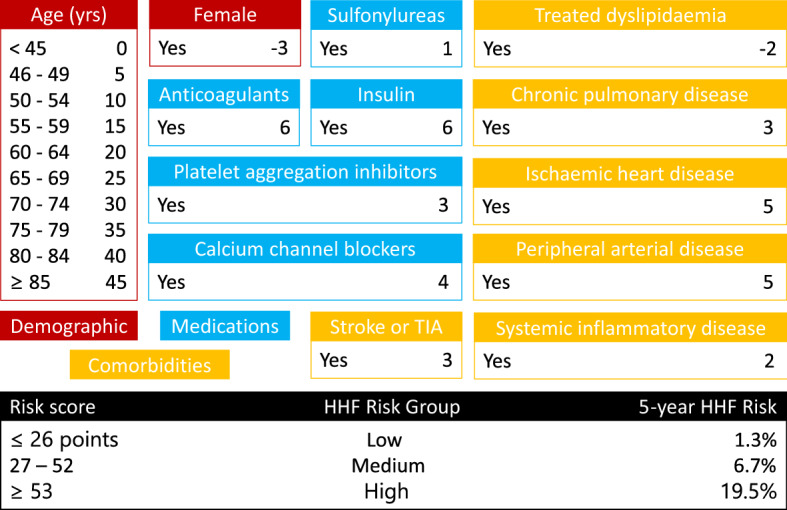
Point-based risk score covariates and corresponding points. Covariates names are reported in red if demographic, blue if medications, and yellow if comorbidities or pre-existing medical conditions. The black table at the bottom of the figure can be easily used to obtain the HHF risk group and the corresponding 5-years HHF risk after computing the patient specific score. The point-based risk score can then be converted into an estimated probability via Eq. (1).

The administrative Cox model’s regression coefficients were used to derive the point-based HHF risk score described in Fig. 3. The score consists of 13 variables: 2 demographic variables, 5 variables related to prescribed medications, and 6 variables related to pre-existing medical conditions or comorbidities. The HHF score can be easily computed by referencing Fig. 3. Specifically, the 5-year HHF score for a single patient is obtained by summing all points related to covariates with a positive answer (Yes), and those attributed to the correct age category. Once the score is computed, the patient-specific HHF risk group can be derived from the black table at the bottom of Fig. 3 (see Supplementary for use-case examples).

Table 2 shows the administrative data-based Cox model’s and point-based risk score’s 1-to-5-year discrimination metrics (AUROC and C-Index) computed on the administrative claims test set. Both the point score and the underlying Cox model exhibited good discrimination ability at all considered PHs. Particularly, the AUROC was > 0.765 for the Cox model and > 0.761 for the HHF score. The C-index confirmed these results with a minimum value of 0.763 (Underlying cox model) and 0.760 (point-based score). Overall, the risk score’s performance was comparable to the one of the underlying administrative Cox model as per the criterion stated in "Discrimination analysis" section. Indeed, the DeLong and Kang tests, respectively, did not detect any statistically significant difference at the 0.05 level. Hence, all subsequent comparisons of discrimination performance were carried out for the simpler, point-based score.

**Table 2:** Administrative point-based score versus cox model discrimination on the administrative test set.

Model	Metric	1 year N events = 132	2 years N events = 258	3 years N events = 389	4 years N events = 503	5 years N events = 627
Administrative point-based HHF score	AUROC	0.788 (0.748–0.827)	0.783 (0.754–0.813)	0.792 (0.769–0.814)	0.777 (0.756–0.798)	0.761 (0.741–0.781)
	C-index	0.786 (0.746–0.825)	0.779 (0.750–0.808)	0.783 (0.761–0.806)	0.770 (0.750–0.791)	0.760 (0.741–0.779)
Underlying administrative Cox model	AUROC	0.789 (0.749–0.829)	0.785 (0.755–0.815)	0.795 (0.772–0.817)	0.781 (0.760–0.802)	0.765 (0.745–0.785)
	C-index	0.787 (0.747–0.827)	0.780 (0.751–0.810)	0.786 (0.764–0.808)	0.773 (0.753–0.794)	0.763 (0.745–0.782)

Discrimination performance of the point-based HHF risk score and the underlying Cox model on the administrative test set. AUROC and C-index are presented together with their 95% CIs. All 15,000 test subjects contributed to the C-index at all PHs, while only 14,051, 12,945, 11,905, 10,744, and 9714 to the 1- to 5- year AUROC; the remaining 949, 2055, 3095, 4226, and 5286 subjects were censored before the respective PH.

#### Discrimination of the administrative, point-based HHF risk score in the diabetes outpatient clinic and primary care test sets

The first row of Table 3 shows the administrative risk score discrimination metrics (AUROC and C-Index) computed on the diabetes outpatient clinic test set. The administrative risk score exhibited good discrimination ability at all considered PHs. Particularly AUROC was > 0.730 and the C-index was > 0.740. The HHF score met the applicability criterion at all PHs and for all metrics, the lowest lower bound of the 95% CIs being 0.620 (> 0.5) for 1-year AUROC.

**Table 3:** Discrimination of point-based score and ad-hoc cox models on the diabetes outpatient clinic and primary care test sets.

Diabetes outpatient clinic test set						
Model	Metric	1 year N events = 9	2 years N events = 21	3 years N events = 31	4 years N events = 42	5 years N events = 56
Administrative point-based HHF score	AUROC	0.754 (0.620–0.887)	0.744 (0.660–0.828)	0.753 (0.681–0.825)	0.730 (0.658–0.802)	0.747 (0.681–0.812)
	C-index	0.756 (0.631–0.882)	0.749 (0.669–0.830)	0.756 (0.689–0.824)	0.740 (0.677–0.803)	0.750 (0.694–0.805)
Diabetes outpatient clinic cox model	AUROC	0.847 (0.741–0.954)*	0.779 (0.677–0.880)	0.779 (0.701–0.857)	0.768 (0.698–0.838)	0.765 (0.703–0.828)
	C-index	0.846 (0.746–0.946)*	0.783 (0.689–0.877)	0.783 (0.709–0.858)	0.775 (0.710–0.841)	0.775 (0.718–0.832)

Primary care test set						
		1 year N events = 2	2 years N events = 6	3 years N events = 9	4 years N events = 14	5 years N events = 20
Administrative point-based HHF score	AUROC	0.761 (0.360–1.00)	0.689 (0.476–0.902)	0.744 (0.600–0.893)	0.770 (0.664–0.877)	0.703 (0.600–0.806)
	C-index	0.762 (0.479–1.00)	0.689 (0.494–0.885)	0.737 (0.592–0.882)	0.758 (0.650–0.866)	0.704 (0.604–0.803)
Primary care cox model	AUROC	0.889 (0.850–0.928)	0.830 (0.744–0.915)	0.848 (0.781–0.916)	0.838 (0.764–0.911)	0.744 (0.644–0.912)
	C-index	0.889 (0.862–0.916)	0.826 (0.747–0.904)	0.842 (0.779–0.906)	0.830 (0.764–0.897)	0.753 (0.666–0.841)

Discrimination performance of the point-based HHF risk score and the ad-hoc Cox models on the diabetes outpatient clinic and primary care test sets. AUROC and C-index are presented together with their 95% CIs. All 1,000 test subjects contributed to the C-index at all PHs for both test sets, while, in the diabetes outpatient clinic test set only 909, 767, 632, 524, and 431 subjects contributed to the 1- to 5- year AUROC; the remaining 91, 233, 368, 476, and 569 subjects were censored before the respective PH. Meanwhile, in the primary care test set, only 909, 838, 760, 683, and 615 subjects contributed to the 1- to 5- year AUROC; the remaining 91, 162, 240, 317, and 385 subjects were censored before the respective PH.

*Highlights statistical significance at the 0.05 level versus the administrative point-based HHF score.

The third row of Table 3 shows the administrative risk score discrimination metrics (AUROC and C-Index) computed on the primary care test set. Much like in the previous case, discrimination was satisfactory with AUROC and C-index > 0.689. Regrettably, partly due to the low short-term incidence of events that led to big confidence intervals, the applicability criterion was only met starting from year 3 (lower bound = 0.592).

#### Discrimination assessment of the administrative, point-based HHF risk score versus ad-hoc cox models on diabetes outpatient clinic and primary care data

The first and second row of Table 3 show the administrative risk score and diabetes outpatient clinic Cox model discrimination metrics (AUROC and C-Index) computed on the diabetes outpatient clinic test set. The discrimination of the model developed specifically for the diabetes outpatient clinic setting, considering also clinical variables, was higher (min AUROC 0.765 vs. 0.730, min C-index 0.775 vs. 0.740); however, the difference (AUROC = 0.093, C-index = 0.09) was statistically significant only in the short term (1 year). This suggests that, in the specialist care context, the variables computable from administrative claims may be as informative as their combination with clinical data in the long term because they capture the general health status of the patient. Instead, clinical-level variables and laboratory biomarkers, appear to be necessary for more accurate short-term predictions, as they are probably more correlated with imminent events.

The third and fourth rows of Table 3 show the administrative risk score and primary care Cox model discrimination metrics (AUROC and C-Index) computed on the primary care test set. Much like in the diabetes outpatient clinic setting, in the primary care, the discrimination of the model developed specifically for this setting, considering also clinical variables, was higher. However, the difference was never statistically significant partly due to the low number of events registered in the test set at 1 and 2 years that led to underpowered comparisons. Despite the HHF score being not applicable at short term in the primary care setting (lower bound of the 95% confidence interval < 0.5 at year 1 and 2), these results confirm what was observed in the diabetes outpatient clinic setting: the point estimate is much higher at short term if clinical-level data and laboratory biomarkers are available, however in the medium-to-long term, their impact on performance is lower as the general health status of the patient, well described by administrative variables, becomes more relevant.

### Calibration analysis

In terms of calibration, the administrative Cox model was good at all years as per a visual inspection of calibration plots, Hosmer–Lemeshow test’s null hypothesis was rejected (*p*-value < 0.05) only at 5 years mostly due to light trends of over/underestimation of HHF probability. Calibration of the point-based risk score was only slightly worse than the one of the original administrative Cox model: calibration plots were acceptable, but the score tended to slightly overestimate HHF probability, leading to the rejection of the Hosmer–Lemeshow test’s null hypothesis at 5 years.

The calibration of the administrative risk score on the diabetes outpatient clinic test set, after performing a linear recalibration, was good and comparable to the one of the diabetes outpatient clinic Cox model, calibration plots were good and the Hosmer–Lemeshow test’s null hypothesis was never rejected.

The calibration of the administrative risk score on the primary care test set, after performing a linear recalibration, was good and better than the one of the primary care Cox model whose calibration plots showed trends of underestimation of HHF probability, and the Hosmer–Lemeshow test’s null hypothesis was rejected at all considered years.

## Discussion and conclusions

In this study, we developed an HHF risk score for patients with T2D based only on administrative claims and assessed its performance across different settings within the same healthcare system. To this end, we analysed three harmonized datasets: (1) administrative claims repository; (2) one diabetes outpatient clinic; (3) a primary care network.

We met the goal of developing and testing an easy-to-use, point-based HHF risk score, using only administrative claims data, that could be reliably applied in the various settings. To the best of our knowledge, this is the first study that proposes a simple point-based HHF risk score for T2D patients considering only claims data. The proposed risk score was applied on data gathered from a diabetes outpatient clinic and a primary care setting to understand whether its performance was still acceptable across different care settings and, finally, it was benchmarked against models developed specifically for the two clinical settings, which included variables not available in the claims database, as possible predictors.

The most important finding of our study was that an HHF risk score based only on claims data preserved its performance when applied to clinical databases. In addition, the claims-based score had comparable performance to clinical data-based scores, especially in the medium-long term. Quite interestingly, the use of clinical-laboratory data to derive the score yielded better performance compared to the claims data only in the short term. This finding highlights that data like blood pressure, BMI, HbA1c, eGFR, and lipids only help in predicting short-term HHF and loose prediction importance in the longer run. This result is consistent with what was observed before^7^ regarding a major adverse cardiovascular event 5-year risk model developed using insurance claims data: the inclusion of variables obtained from laboratory measurements as well as administrative variables only marginally improved performance.

Age was arguably the most important feature for all developed models, as it was immediately selected by FRFS in all contexts. The variable sex, another feature known to be strongly related to outcome^37^, was selected only in the administrative setting but neither in the diabetes outpatient clinic nor in the primary care settings. There, the information brought about by sex is probably incorporated in other clinical variables, such as eGFR (whose calculation is sex-specific) and variables with marked sex-based differences, like BMI and lipids clinical variables.

The highest discrimination was achieved at year 3 for all developed models most likely due to the combined effect of (A) predictive power naturally declining, and (B) event incidence naturally increasing as the prediction horizon extends. The discrimination of the point-based risk score was compared to the one of the underlying Cox model considering data of 15,000 patients belonging to the administrative test set. As diabetes prevalence in Italy is around 6%, they correspond to the expected portion of patients with diabetes living in e.g., a city of 250,000 inhabitants. The point-based risk-score achieved good discrimination and it was equivalent to the one of the administrative Cox model; calibration was acceptable but, expectedly, slightly worse. Performance losses were expected when converting Cox models into point-based scores for two main reasons: (1) continuous variables must be divided into clinically significant categories, and (2) Cox regression coefficients, which are typically real values, must be approximated to the nearest integer. Specifically, in this study, the main driver of performance loss was likely age categorisation: since age was the strongest among all predictors, reducing its variability via binning was bound to affect the resolution of the output. Indeed, while discrimination did not suffer significant losses, calibration was penalised by the restriction of the number of possible predicted probability values to 88, i.e., the number of possible different values for the point-based score. The HHF risk score proved applicable in the medium-to-longer term to the diabetes outpatient clinic ($PH\ge 2$ years) and primary care settings ($PH\ge 3$ years), where it retained comparable performance. These encouraging results are mainly to be attributed to the sample size of the administrative claims dataset used for development, which is noticeably larger than a typical clinical dataset.

Notable aspects of this study include the availability of three datasets collected within the same healthcare system where caregiving is equally distributed, and the sample size of the administrative claims dataset, which is among the largest Italian administrative datasets and well represents the diabetic population of the Veneto region. Particularly, the availability of three datasets coming from such different contexts enabled the definition of a point-based score that can be easily applied not only at the institutional level (where it was developed), but also, after computing the relevant variables, directly by physicians.

A strength of the proposed score is that it maintained good performance even though it was developed considering the most challenging scenario, i.e., predicting HHF in patients who had no evidence of previous or latent HF. Indeed, not only were patients with known, pre-existing HF excluded from the analysis (a common practice when developing risk scores^11^), but further exclusion criteria were also set based on specific patterns of diuretics usage strongly correlated with suspect or latent HF^38^.

The main limitation of this study is that the three databases were not separated as the administrative dataset comprises all registered diabetic patients of the Veneto Region. An intersection between the diabetes outpatient clinic and the primary care dataset is less likely as the first was collected at a single clinic whilst the latter was patchy sampled across the entire region. However, the same patient is very likely to have a different representation in the various datasets as the index date differs. The anonymisation of the datasets and the independent data gathering procedure performed for the three datasets led to the impossibility of assessing the magnitude and effect of patient intersections among the three datasets. Another possible limitation is the low number of events recorded in the primary care dataset as it may lead to unstable results. However, considering that diabetic patients followed by their general practitioner are typically healthier and have fewer complications, a lower HHF frequency is reasonable and expected in this setting.

Only the Cox model was considered as identifying the best performing methodological approach was not among the aims of this study. In fact, our focus was on providing a simple, yet effective, risk-score that could be easily used in everyday clinical practice. Future studies may focus on development, benchmarking, and interpretability of more complex black box approaches, such as deep learning.

In conclusion, we developed a risk score for HHF occurrence in T2D based solely on administrative claims that can be used by general practitioners, diabetes specialists, and the healthcare governance. Incorporating less readily available clinical-laboratory data yielded better performance only in the short term. Therefore, the output of this study can help projecting resource allocation on the medium-long range and is instrumental to optimization of healthcare delivery.

## Supplementary Information

Supplementary Information.

## Data Availability

The datasets generated during and/or analysed during the current study are not publicly available as they are owned by the Regional healthcare system and SVEMG and were used under license for the current study. Data are however available from the authors upon reasonable request and with permission of the Regional healthcare system and SVEMG.
