# Supplementary material for: Performance assessment across different care settings of a heart failure hospitalisation risk-score for type 2 diabetes using administrative claims
Source: Sci Rep. 2022 May 11;12:7762. doi: 10.1038/s41598-022-11758-9 (PMC9095603; doi:10.1038/s41598-022-11758-9)
Supplement: Supplementary file 1 — Supplementary Information. [file 41598_2022_11758_MOESM1_ESM.pdf]

Performance Assessment Across Different Care Settings of a Heart Failure Hospitalisation Risk-Score for Type 2 Diabetes Using Administrative Claims

Alessandro Guazzo[1], Enrico Longato[1], Giovanni Sparacino[1], Mario Luca Morieri[2] Bruno Franco-Novelletto[3,4], Maurizio Cancian[3,4], Massimo Fusello[3], Lara Tramontan[5], Alessandro Battaggia[3,4, ⱡ], Angelo Avogaro[2, ⱡ], Gian Paolo Fadini[2,ⱡ], and Barbara Di Camillo[1,6,ⱡ,*]

[1] University of Padova, Department of Information Engineering, Padova, 35122, Italy
[2] University of Padova, Department of Medicine, Padova, 35128, Italy
[3] Scuola Veneta di Medicina Generale (SVEMG), Italy
[4] Società Italiana di Medicina Generale e delle Cure Primarie (SIMG), Italy
[5] Arsenàl.IT, Veneto's Research Centre for eHealth Innovation, 31100 Treviso, Italy
[6] University of Padova, Department of Comparative Biomedicine and Food Science, Legnaro (PD), 35020, Italy
[*] barbara.dicamillo@unipd.it
[ⱡ] these authors contributed equally to this work

HHF risk-score use case example

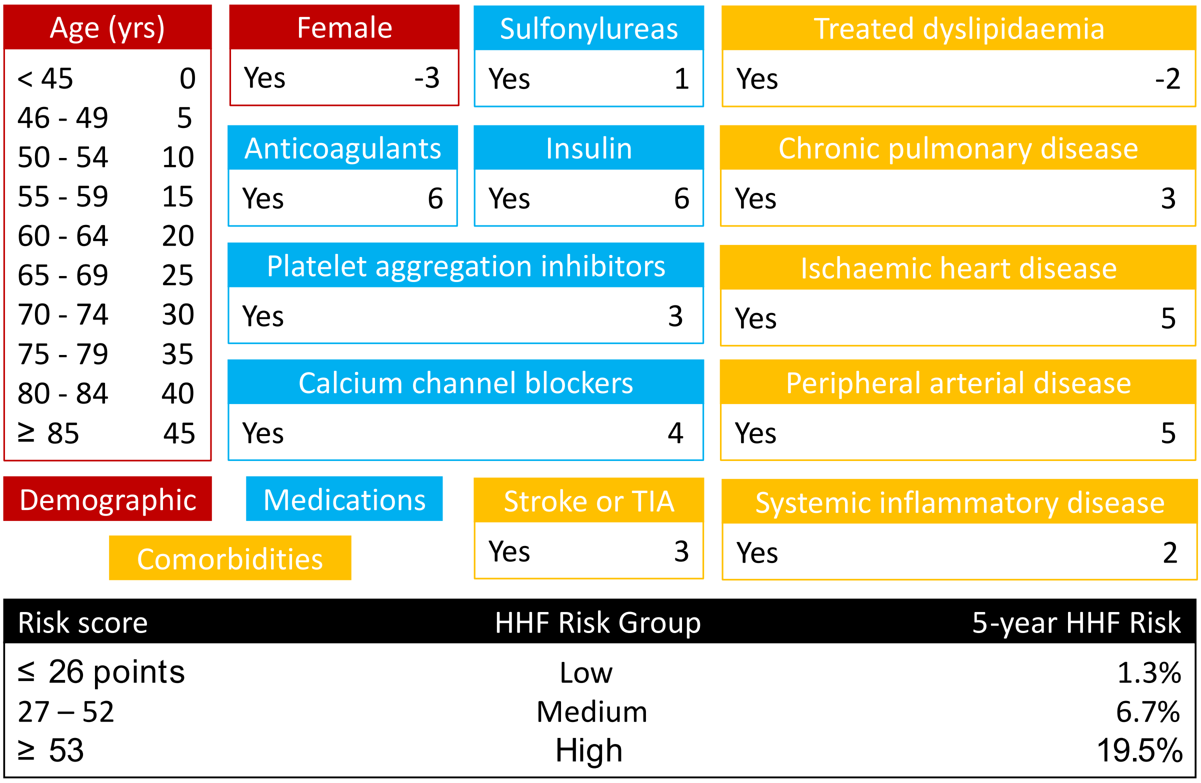

Supplementary Figure 1: Point-based risk score covariates and corresponding points. Covariates names are reported in red if demographic, blue if medications, and yellow if comorbidities or pre-existing medical conditions. The black table at the bottom of the figure can be easily used to obtain the HHF risk group and the corresponding 5-years HHF risk after computing the patient specific score.

To illustrate the use of the proposed point-based heart failure hospitalisation risk score described in the figure above, consider a 67-year-old Male patient that suffers from type 2 diabetes treated with insulin, sulfonylureas,

anticoagulants, and calcium channel blockers, with a previous medical history of ischaemic heart disease, peripheral heart disease and systemic inflammatory disease.

The patient specific HHF risk score can be easily computed by summing all points related to covariates with a positive answer (Yes), and those attributed to its age category:

$$\begin{aligned} HHF\ risk\ score &= 6^{th} Age\ category + Insulin + Sulfonylureas + Anticoagulants + Calchium\ channel\ blockers \\ &+ ischaemic\ heart\ disease + peripheral\ arterial\ disease + Systemic\ inflammatory\ disease \\ &= 25 + 6 + 1 + 6 + 4 + 5 + 5 + 2 = 54 \end{aligned}$$

Then, the HHF risk group and the corresponding 5-year HHF risk of our considered patient can be obtained from the black table at the bottom of the figure:

$$HHF\ risk\ score = 54 \rightarrow High\ HHF\ risk\ group \rightarrow 19.5\%\ \ probability\ of\ HHF\ within\ 5\ years$$

The considered patient is thus at high 5-year HHF risk as 19.5% of patients associated to the same risk group, according to the proposed point-based risk score, developed an HHF within 5 years.

To give another use example consider a 56-year-old Female patient that suffers from type 2 diabetes treated with insulin, platelet aggregation inhibitors and with a treated dyslipidaemia.

The patient specific HHF risk score can be easily computed by summing all points related to covariates with a positive answer (Yes), and those attributed to its age category:

$$\begin{aligned} HHF\ risk\ score &= 4^{th} Age\ category + Female + Insulin + Platelet\ aggregation\ inhibitors + Treated\ dyslipidaemia \\ &= 15 - 3 + 6 + 3 - 3 = 18 \end{aligned}$$

Then, the HHF risk group and the corresponding 5-year HHF risk of our considered patient can be obtained from the black table at the bottom of the figure:

$$HHF\ risk\ score = 18 \rightarrow Low\ HHF\ risk\ group \rightarrow 1.3\%\ \ probability\ of\ HHF\ within\ 5\ years$$

The considered patient is thus at low 5-year HHF risk as 1.3% of patients associated to the same risk group, according to the proposed point-based risk score, developed an HHF within 5 years.

Supplementary Table SI
Set of Possible Predictors Characteristics of the Administrative Dataset

	**Training Set**	**Validation Set**	**Test Set**
**N. subjects**	146,018	15,000	15,000
**Age (years)**	67.6 ± 11.5	67.6 ± 11.5	67.6 ± 11.5
**Female sex**	60,755 (41.6%)	6,251 (41.7%)	6,306 (42.0%)
**Diabetes duration (years)**	6.1 ± 5.4	6.1 ± 5.4	6.1 ± 5.4
**Cancer**	17,043 (11.7%)	1,682 (11.2%)	1,707 (11.4%)
**Anaemia**	865 (0.6%)	106 (0.7%)	88 (0.6%)
**Peripheral arterial disease**	730 (0.5%)	96 (0.6%)	73 (0.5%)
**Chronic kidney disease**	1,064 (0.7%)	119 (0.8%)	108 (0.7%)
**Chronic pulmonary disease**	17,956 (12.3%)	1,898 (12.7%)	1,851 (12.3%)
**Dyslipidaemia**	77,222 (52.9%)	7,873 (52.5%)	7,895 (52.6%)
**Complications renal**	98 (0.07%)	4 (0.03%)	11 (0.07%)
**Infarction**	1,869 (1.3%)	205 (1.4%)	175 (1.2%)
**Ischemic heart disease**	9,978 (6.8%)	1,031 (6.9%)	1,025 (6.8%)
**Stroke or TIA**	3,362 (2.3%)	382 (2.5%)	330 (2.2%)
**Systemic inflammatory disease**	2,299 (1.6%)	228 (1.5%)	253 (1.7%)
**Calcium channel blockers**	34,840 (23.9%)	3,507 (23.4%)	3,531 (23.5%)
**Beta blockers**	38,310 (26.2%)	4,059 (27.1%)	3,917 (26.1%)
**Acarbose**	2,265 (1.6%)	234 (1.6%)	207 (1.4%)
**Dpp4i**	11,973 (8.2%)	1,201 (8.0%)	1,226 (8.2%)
**ACE inhibitors**	92,694 (63.5%)	9,503 (63.4%)	9,422 (62.8%)
**Insulin**	26,455 (18.1%)	2,840 (18.9%)	2,683 (17.9%)
**Anticoagulants**	14,258 (9.8%)	1,432 (9.5%)	1,396 (9.3%)
**Pioglitazone**	8,069 (5.5%)	822 (5.5%)	845 (5.6%)
**Ezetimibe**	1,301 (0.9%)	120 (0.8%)	122 (0.8%)
**Sulfonylureas**	58,350 (40.0%)	6,027 (40.2%)	5,923 (39.5%)
**Metformin**	115,488 (79.1%)	11,769 (78.4%)	11,875 (79.2%)
**Platelet aggregation inhibitors**	55,769 (38.2%)	5,764 (38.4%)	5,679 (37.9%)
**HHF**	6,473 (4.4%)	673 (4.5%)	712 (4.7%)

Continuous variables are presented as mean ± standard deviation, binary variables as count (percentage relative to N. subjects).
